# Alcohol solvothermal reduction for commercial P25 to harvest weak visible light and fabrication of the resulting floating photocatalytic spheres

**DOI:** 10.1038/s41598-019-50457-w

**Published:** 2019-09-25

**Authors:** Ting Wang, Yao Li, Jia-hao Pan, Yan-ling Zhang, Li-guang Wu, Chun-ying Dong, Chun-juan Li

**Affiliations:** School of Environmental Science & Engineering, Zhejiang Gongshang University, Hangzhou, 310012 China

**Keywords:** Environmental chemistry, Nanoscale materials

## Abstract

In this study, to fabricate stable floating photocatalytic spheres, facile alcohol solvothermal reduction was first employed to modify commercial TiO_2_ (P25) photocatalysts to harvest visible light and improve their performances for photodegrading phenol in seawater exciting by visible light. Floating photocatalytic spheres were then prepared by loading reduced P25 photocatalysts on inner and outer surfaces of acrylic hollow spheres. The structural characterizations showed that reduction of P25 introduced disorder–crystalline shell–core structures with present Ti^3+^ in reduced P25 photocatalysts. These features facilitated visible light response and phenol degradation in seawater under visible light irradiation. As reduction time or temperature of alcohol solvothermal process rose, more Ti^3+^ and shell–core structures were introduced into reduced P25, resulting in higher performances towards phenol degradation in seawater. However, extended periods of time and elevated temperatures decreased disordered layer of reduced P25, deteriorating the photocatalytic performances. Thanks to good light transmission of the hollow spheres and the high performance of the reduced P25, the photocatalytic performances of spheres loaded with reduced P25 could effectively degrade phenol in seawater even at low concentrations. The removal rate of phenol by floating spheres reached more than 95% after 8 h. In addition, the floating spheres displayed good stability and convenient reusability after six repeated photocatalytic degradation for phenol in seawater, promising features for future treatment of organic pollutants in oceans.

## Introduction

The rapid development of environmental science and technology has increased awareness regarding environmental pollution^1–3^. For example, concentration levels of organic pollutants (e.g.; benzene–based organic pollutants) in discharged wastewaters corresponding to only the chemical oxygen demand (COD) value could result in accumulation of refractory organic pollutants for long periods of time^4,5^. Though concentrations of organic pollutants in wastewaters meet the requirements for emission standards, their refractory properties would cause accumulation in water bodies and lead to serious environmental pollution^4,5^. For instance, the discovery of polycyclic aromatic hydrocarbons (PAHs) in oceans is one noteworthy global environmental issue^6,7^. The accumulation of aromatic organic pollutants in land drainage wastewaters and their transfer to oceans generate PAHs in seawater under long–term photochemistry. PAHs are currently been detected in many sea areas around the world, and even in deep–sea fish, becoming serious global environmental pollution problem^6,7^. To overcome pollutants issues, the policy requirements for both organic pollutant emission and control have been reinforced. However, current existing aromatic organic pollutants in oceans require urgent removal. Traditional wastewater treatment technologies, such as adsorption, microbial treatment and membrane separation are inadequate due to the extremely low concentrations of organic pollutants in vast marine and complex seawater systems^8,9^. Alternatively, TiO_2_–based heterogeneous semiconductor photocatalysts are promising for degradation of highly toxic organic pollutants at low levels in seawaters thanks to their low energy consumption non–selective degradation, and pollution–free process^10–12^.

However, powder photocatalysts suffer from low stability during heterogeneous photocatalytic degradation of organic pollutants in seawater. On the other hand, TiO_2_ nanoparticles with sizes less than 100 nm could effectively degrade organic pollutants under UV light irradiation^10–12^. Some studies suggested that quantum–sized TiO_2_ particles with less than 10 nm in size have excellent photocatalytic degradation performances^10–12^. Nevertheless, small nanoparticles could easily agglomerate during preparation to form larger particles with low catalytic activities. The most potential and concerned commercial TiO_2_ photocatalyst is P25 from Evonik Group (Formerly Degussa)^13,14^. Its increased photocatalytic activity is due to the rutile structure as an electron sink to hinder recombination of electron-hole pairs and allow holes Migration to the surface. P25 is also stable when used as a promising photocatalyst for wastewater treatments^13,14^. However, two challenges remain to be solved before using P25 photocatalysts for removal of aromatic organics from seawater. The first one is how to improve convenient recyclability for recovery of catalyst. Some studies reported powder photocatalysts like P25 as an active component to be loaded on carriers or directly generated TiO_2_ particles on carrier surface^15,16^. Lightweight materials floating on water surface, such as perlite, expanded graphite and porous glass microspheres are attractive carriers. These supported photocatalysts floating on water could be prepared by loading powder photocatalysts or generating TiO_2_ on their surfaces with very simple recyclability features. Second, high visible light utilization is required for carriers and loaded active components since natural light source in marine consists of visible light source with less than 1 mW·cm^−2^ ^17,18^. However, most floating carriers have shown low visible light transmittance, inadequate for practical applications. In addition, the application of floating supported photocatalysts to remove organic pollutants in seawater has not been reported in other literature.

In this study, to expand the photocatalytic removal of aromatic organic pollutants in seawater, P25 was first surface modified by alcohol solvothermal reduction to enhance its visible light response^19–21^. The modified P25 photocatalysts showed elevated photocatalytic performances towards phenol degradation in seawater due their high visible light responses. Next, transparent hollow acrylic spheres with thin walls were employed as carriers to load the modified P25 photocatalysts through treatment by silane coupling agent. The combination between fully transparent thin-walled hollow acrylic spheres with high performance modified P25 photocatalysts induced floating photocatalytic spheres with more enhanced photocatalytic degradation properties towards phenol degradation even at low concentrations in seawater. Furthermore, good stability during repeated photocatalytic degradation experiments was observed with the novel material, promising for treatment of organic pollutants in oceans.

## Experimental

### Materials

Gas nanometer titanium dioxide without porous (TiO_2_) P25 (21 nm, 50 m^2^·g^−1^) was obtained from Degussa, Germany. Analytical grade absolute ethanol was purchased from Reagent Chemical Manufacture (Shanghai) and was distilled then stored over 4 Å molecular sieves prior to use. Sodium chloride (NaCl), magnesium chloride (MgCl_2_), sodium sulfate (Na_2_SO_4_), calcium chloride (CaCl_2_) and phenol were all of analytical grade and purchased from Sinopharm Chemical Reagent Co.; Ltd (Shanghai, China). γ–Methacryloxypropyltrimethoxysilane (KH-570) was obtained from Reagent Chemical Manufacturing (Shanghai, China). Transparent acrylic hollow spheres with 5 cm in diameter and 5 mm in wall thickness were provided by Shenzhen Haotian Plexiglass Company (Fig. S1, Supplementary Material).

### Reduction of P25 photocatalysts

First, 1.0 g of TiO_2_ powders (P25) and 120 mL of absolute ethanol were added into a Teflon–covered stainless steel autoclave. After 15 min ultrasonic treatment(KQ-300TDE; 300 W, 80 kHz), TiO_2_ dispersed well in absolute ethanol. The autoclave was then placed in Electric thermostaticdrying oven for solvothermal treatment by ethanol at 170 °C for different time periods (Table 1). After several centrifugation–washing cycles, reduced P25 photocatalysts were obtained.

**Table 1:** Reduction conditions of different photocatalysts.

Catalyst	Temperature in solvothermal treatment/°C	Reduction time/h
P25	—	—
P25-170-3	170	3
P25-170-6	170	6
P25-170-12	170	12
P25-170-24	170	24

For comparison, reduction for P25 using High purity hydrogen under high hydrogen pressure was performed for 20 days referring to the literature^19^. The obtained hydrogen-reduced P25 was denoted as P25-H_2_.

### Preparation of floating photocatalytic spheres

Four transparent acrylic hollow spheres were immersed in 450 mL ethanol and 50 mL water then ultrasonically treated using KQ-300TDE probe for 10 min at 300 W and 80 kHz. Next, 0.5 g coupling agent (KH570) was added and sonicated for 30 min.

Afterwards, 0.5 g of as-prepared reduced P25 and 0.10 g KH570 were dispersed in 50 ml of mixed solution containing 45 mL ethanol and 5 mL water. The mixture was sonicated at 30 °C for 1 h. The suspension containing P25 powder was then added to the acrylic hollow sphere’s suspension. Next, P25 powder was completely loaded on the inner and outer surfaces of the spheres for 5 h with the help of KH570 to yield the photocatalytic floating spheres after drying. The hollow spheres could float steadily on simulated seawater surface (Fig. S2).

### Characterization

The morphologies of the particles were viewed and their corresponding selected areas of electron diffraction (SAED) were obtained by transmission electron microscopy (TEM, JEM-2010CX). The changes in the TiO_2_ crystallization in reduced P25 were evaluated by X-ray diffraction (XRD, D/max-rA XRD instrument XD-98) with Cu Kα radiation (1.5406 Å). X-ray photoelectron spectroscopy (XPS, Thermo ESCALAB 250, USA) with Al Kα X-ray (hm = 1486.6 eV) radiation operating at 150 W was utilized for surface properties analysis. Structure and phase transformations in nanophase TiO_2_ were investigated by Renishaw Invia Raman spectrometry (US). He-Ne laser at 532 nm was used as radiation light source. The X-band electron paramagnetic resonance (EPR) spectra were recorded on a Bruker EMX spectrometer equipped with cylindrical quart tube operating at 100 kHz field modulation and temperature of 100 K.

Photo–electrochemical analyses were performed on a CHI660E workstation using standard three-electrode configuration in 0.5 M Na_2_SO_4_ electrolyte. The working electrode was prepared by deposition of TiO_2_/Ag film on FTO glass. A Pt sheet was used as counter electrode and saturated calomel electrode (SCE) as reference. The UV–visible diffuse reflectance spectra (DRS) were obtained on UV–vis spectrophotometer (UV–vis DRS: TU-1901, China) equipped with integrating sphere assembly using BaSO_4_ as reflectance sample. UV–vis spectrophotometry (UV–vis DRS: TU-1901, China) was utilized to measure changes in phenol concentration at wavelength of 510 nm.

### Photodegradation for phenol in seawater by different photocatalysts

Simulated seawater composed of 2.5% NaCl, 1.1% MgCl_2_, 0.40% Na_2_SO_4_ and 0.16% CaCl_2_ dissolved in distilled water without CO_2_ was used as seawater^22,23^. Photodegradation of phenol at 5.0 mg·L^−1^ in seawater was employed to test the performances of different catalysts under visible light irradiation^24^. A 30 W LED–lamp with 400 nm cut–off filter was used as visible light source. Prior to irradiation, each suspension was stirred in the dark for 30 min to ensure establishment of adsorption/desorption equilibrium. Next, sampled suspensions were centrifuged, and the upper clear solutions were extracted at 30 min intervals. UV–vis spectrophotometry was utilized to measure changes in phenol concentration at wavelength of 510 nm using the 4-aminoantipyrine spectrophotometric method^25,26^.

### Photocatalysis of phenol in seawater using floating spheres photocatalysts

**Figure 1:**
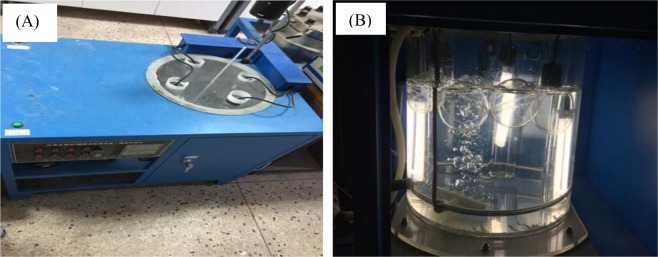
(**A**) The photocatalytic device of floating photocatalytic spheres. (**B**) Images of photodegradation process by floating spheres under visible light irradiation.

The photocatalytic reaction device under visible light source was composed of four 15 W fluorescent lamps with visible light source and 10 L artificial seawater containing different initial concentrations of phenol (as shown in Fig. 1), in which air was introduced by bubbling during photodegradation. UV–vis spectrophotometry was also employed to follow changes in phenol concentration at wavelength of 510 nm and 30 min intervals using the 4-aminoantipyrine spectrophotometric method^25,26^.

## Results and Discussion

### Morphology and structure of reduced P25

The FTIR spectra of different reduced P25 photocatalysts by alcohol solvothermal process are depicted in Fig. S3. For comparison, the FTIR spectra of pristine P25 and P25-H_2_ are shown in Fig. S3. The hydrogenation reduction or alcohol solvothermal process significantly weaken the absorption peaks owing to oxygenated functional groups on P25 surface. This confirmed that reduction of P25 also occurred during alcohol solvothermal process, similar to reduction of P25 by hydrogenation. Furthermore, both alcohol reduction in solvothermal process and hydrogenation reduction could remove oxygenated functional groups on P25 surface^27,28^. On the other hand, the absorption peaks of oxygenated functional groups on P25-H_2_ were the weakest among other reduced P25 photocatalysts due to strong hydrogenation reduction. The reduction of P25 during alcohol solvothermal process was confirmed by gas chromatography (GC), analyzing changes in alcohol solvent before and after the solvothermal process (Figs S3–S8 and Tables S1–5). It will be noted that few acetaldehydes were generated in alcohol after solvothermal process. As reduction time increased, the content of acetaldehyde also rose.

**Figure 2:**
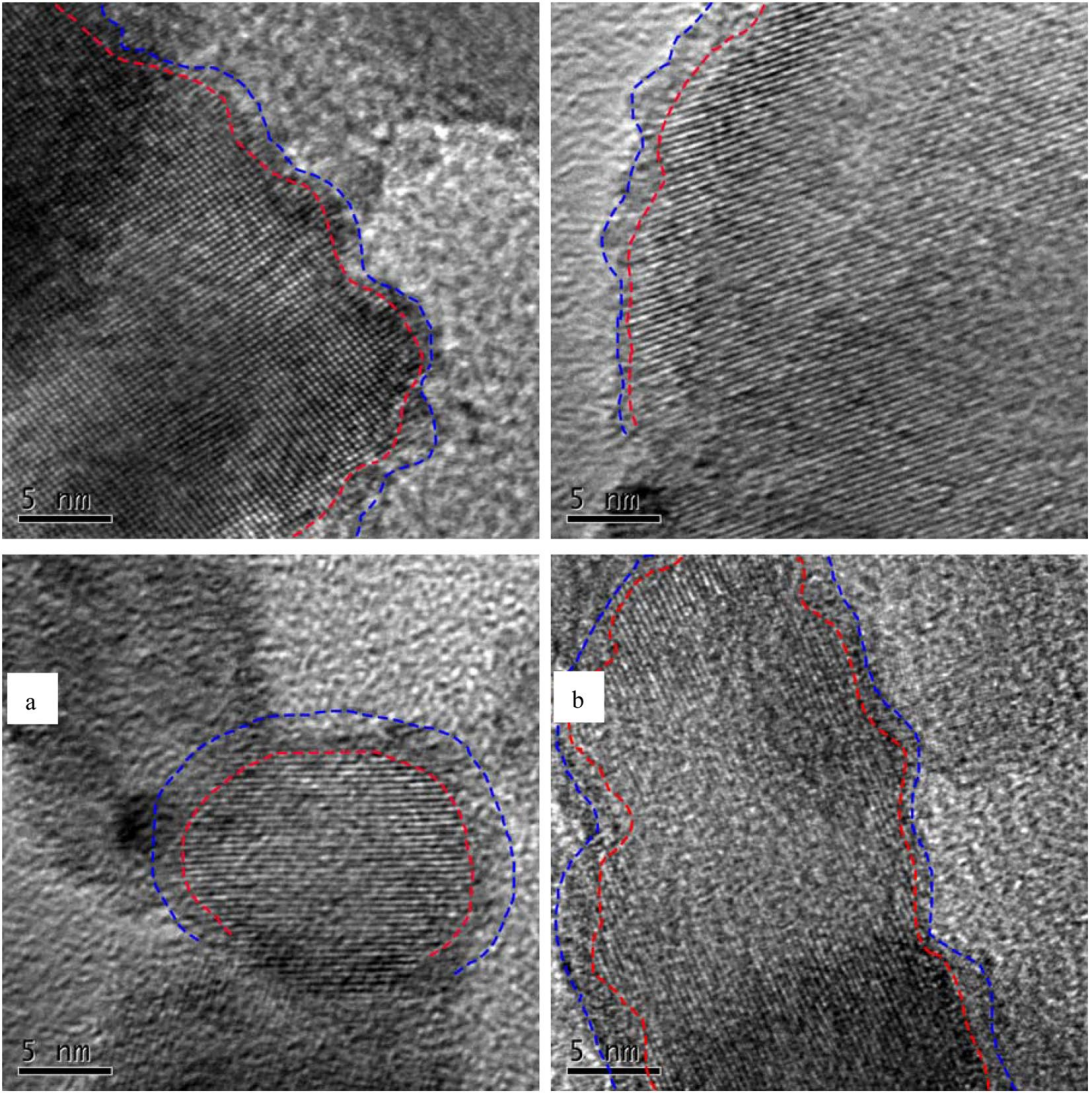
HRTEM images of different reduced P25 photocatalysts: (**a**) P25-170-3, (**b**) P25-170-6, (**c**) P25-170-12, and (**d**) P25-170-24.

The TEM images of different reduced P25 are shown in Fig. S9. No significant differences in morphology between photocatalysts were observed. The hydrogenation and alcohol solvothermal reductions slightly changed the morphologies of most reduced P25 powders, since commercial P25 had excellent stability. However, the HRTEM images in Figs 2 and S10 revealed obvious differences between the crystalline structures of reduced P25 and pristine P25. There are well-resolved lattice features in HRTEM image of pristine P25 (Fig. S10), suggesting that pristine P25 composes of highly crystallized TiO_2_ particles. As shown in Fig. S11, the hydrogeneration reduction caused formation of an evident disorder–crystalline shell-core structure on P25-H_2_ surface, consistent with literature^19–21^. In addition to disorder–crystalline structure on surface, the strong hydrogenation reduction could also destroy all crystalline of some whole TiO_2_ particles. In Fig. S11, some whole TiO_2_ particles exhibited the morphology as amorphous TiO_2_ structures.

Different reduced P25 photocatalysts by alcohol solvothermal process showed thinner disorder layer on P25 surface when compared to the layer deposited on P25-H_2_ surface, further confirming the reduction of P25 during alcohol solvothermal process. As reduction time increased from 3 h to 12 h, the disordered layer on reduced P25 surface became more obvious. By comparison, the disordered layer on reduced P25 surface after solvothermal reduction for 24 h appeared thinner than that on P25-170-12 surface. This may be due to continuous solvothermal heat treatment of P25, transforming some disordered amorphous TiO_2_ to crystalline TiO_2_ after reduction of most P25 surface.

**Figure 3:**
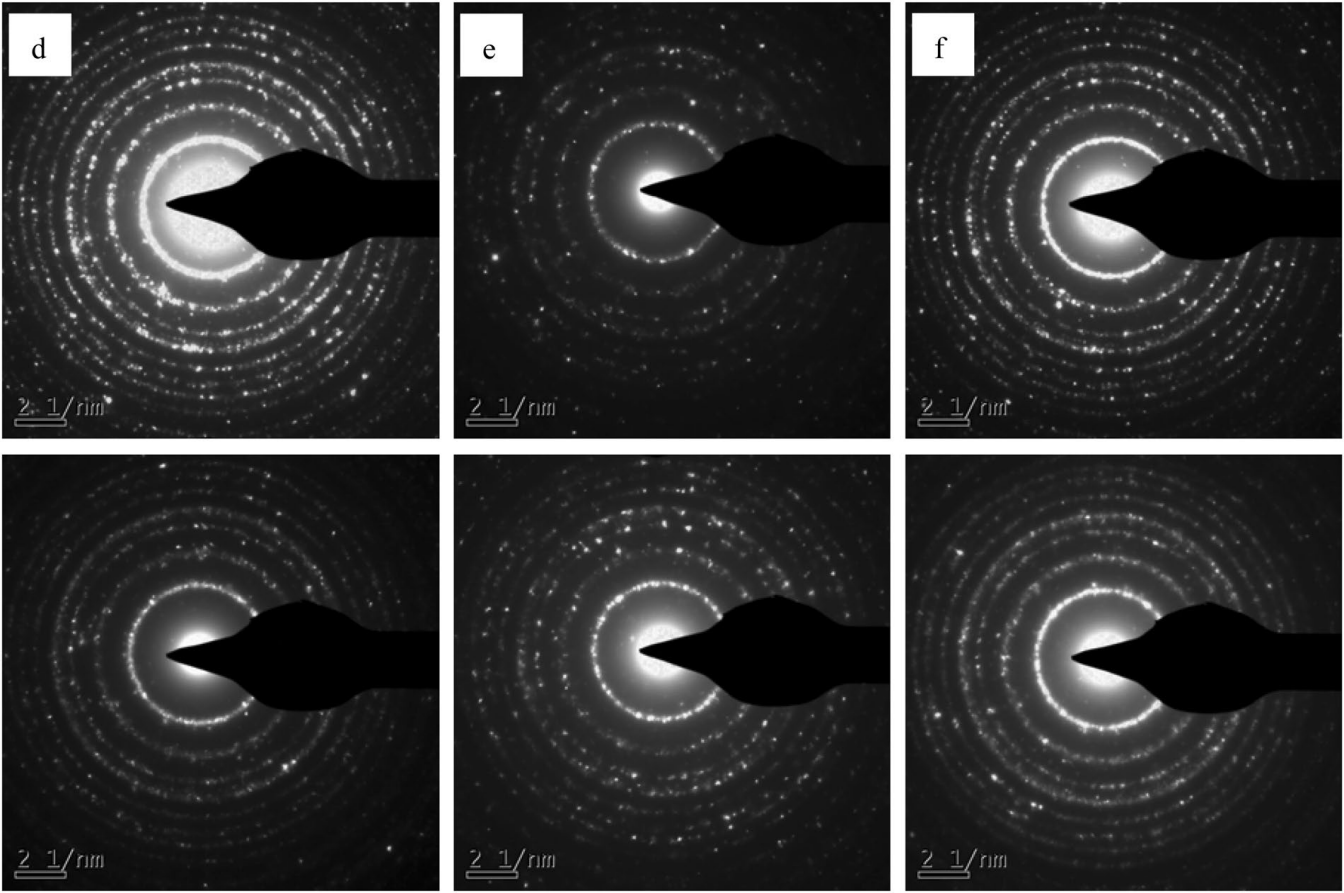
HRTEM images of pristine P25 and different reduced P25 photocatalysts: (**a**) pristine P25, (**b**) P25-H_2_, (**c**) P25-170-3, (**d**) P25-170-6, (**e**) P25-170-12, and (**f**) P25-170-24.

The effects of alcohol solvothermal reduction on crystal structure of TiO_2_ in P25 were further confirmed by SAED and XRD. As shown in Fig. 3, the SAED profile of pristine P25 suggested highly crystalline TiO_2_ with rutile and anatase crystal structures^29^. Both hydrogeneration reduction and alcohol solvothermal destroyed the crystalline TiO_2_ to form amorphous TiO_2_ on reduced P25 surface. Thus, the diffraction rings in SAED patterns of reduced P25 became obviously faint when compared to that of pristine P25. The strong hydrogeneration reduction induced P25-H_2_ with faintest diffraction rings. These results were consistent with HRTEM images. By comparison, reduced P25 by alcohol solvothermal process illustrated P25-170-12 with faintest SAED diffraction rings. However, the SAED diffraction rings of P25-170-24 became clear, confirming recrystallization of some amorphous TiO_2_ in P25-170-24.

**Figure 4:**
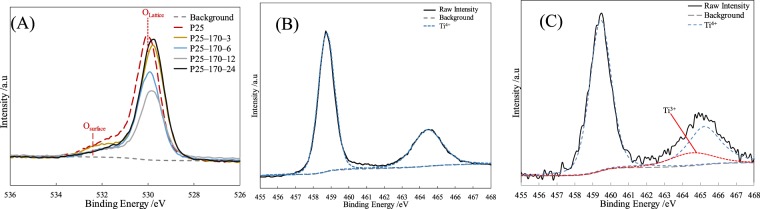
(**A**) XPS profiles of O1s in different photocatalysts, (**B**) Ti2p in pristine P25, and (**C**) Ti2p in P25-170-12.

The XRD peaks of pristine P25 and all reduced P25 (as shown in Fig. S12, SI) could be well indexed to anatase TiO_2_ (JCPDS NO. 21–1272) and rutile TiO_2_ (JCPDS NO. 21–1276)^30^. In addition, reduction for P25 weakened the crystal peaks of TiO_2_, where strongrt reduction reflected more evident weakening effect on crystal peaks. On the other hand, no other crystal peaks were observed except those of TiO_2_. In Fig. S13, XRD data showed only presence of three elements consisting of C, O and Ti in pristine P25 and all reduced P25. This was confirmed by XPS of all P25 photocatalysts. The High–resolution XPS profiles of O1s and Ti2p in all catalysts and their corresponding deconvolution results are listed in Figs 4 and S14. According to literature and Fig. 4(A), XPS profiles of O1s indicated existence of two chemical states of oxygen in all catalysts: crystal lattice oxygen and non–lattice oxygen^31^. The above characterizations suggested that reduced P25 photocatalysts did contain crystalline structures except for crystalline TiO_2_. Thus, lattice oxygen could mainly be attributed to presence of oxygen in lattice structure of anatase TiO_2_ and rutile TiO_2_, whereas non–lattice oxygen originated from oxygenated functional groups like hydroxyl groups on the catalyst surface. In Fig. 4(A), the alcohol solvothermal reduction declined peak intensities of both crystal lattice oxygen and non–lattice oxygen. The decrease in non–lattice oxygen must be due to removal of some oxygenated functional groups from the catalyst surface. Interestingly, the crystal lattice oxygen in reduced P25 was obviously smaller than that in P25. The later could be attributed to formation of oxygen vacancy in reduced P25 after alcohol solvothermal reduction, accompanied by generation of amorphous TiO_2_. As reduction time increased from 3 h to 12 h, the decrease in crystal lattice oxygen became more obvious, indicating formation of more oxygen vacancies in reduced P25. However, recrystallization of some amorphous TiO_2_ in P25-170-24 raised the peak intensity of crystal lattice oxygen. In addition, all peaks in O1s XPS profiles slightly shifted to lower binding energies after the alcohol solvothermal reduction.

The XPS profile for Ti2p in pristine P25 showed two peaks at approximately 464.3 and 458.5 eV (Fig. 4(B)), attributed to Ti_2p1/2_ and Ti_2p3/2_ of Ti^4+^ and suggesting existence of Ti as Ti^4+^ in pristine P25^32,33^. For different reduced P25 photocatalysts (Figs 4(C) and S14), a new peak appeared at approximately 462.5 eV, corresponding to Ti_2p1/2_ of Ti^3+^ in catalysts. This was induced by reduction of Ti^4+^ to Ti^3+^ on P25 surface accompanied by introduction of disorder–crystalline shell-core structure^32,33^.

**Figure 5:**
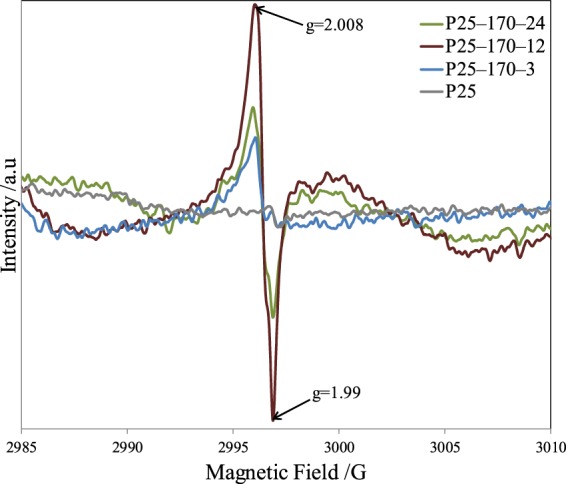
EPR spectra of pristine P25 and different reduced P25 photocatalysts.

The formation of oxygen vacancies and introduction of Ti^3+^ into reduced P25 photocatalysts were further supported by EPR spectroscopy (Fig. 5). The obvious signals of reduced P25 photocatalysts observed at g = 2.008 were associated with oxygen vacancies, and those at g = 1.99 observed in reduced P25 photocatalysts were attributed to Ti^3+^ sites. The changes in EPR signals were consistent with XPS analysis^34,35^.

**Figure 6:**
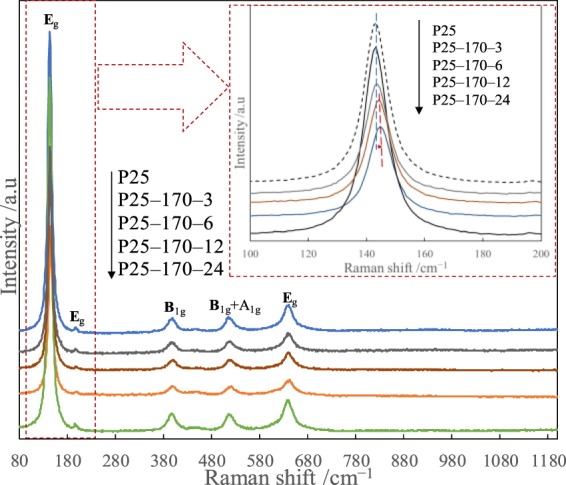
Raman spectra of pristine P25 and different reduced P25 photocatalysts.

Raman spectroscopy is a powerful technique for structural identification, hence was employed to examine the structural characteristics and crystal deficiency of reduced P25 photocatalysts (Fig. 6). A total of five characteristic Raman active modes (3E_g_ + 2B_1g_ + A_1g_) with frequencies at about 141, 193, 387, 505 and 626 cm^−1^ were obtained, belonging to typical anatase Raman bands^36,37^. Compared to Raman spectra of pristine P25, *E*_*g*_ mode of reduced P25 obviously shifted and its linewidth broadened. This could be ascribed to lattice disorder or localized defects associated with oxygen vacancies^36,37^. For P25-170-24, its five peaks looked similar to those of pristine P25, confirming enhanced crystallinity of TiO_2_ after reduction time of 24 h.

### The expanding of visible light response of reduced P25

**Figure 7:**
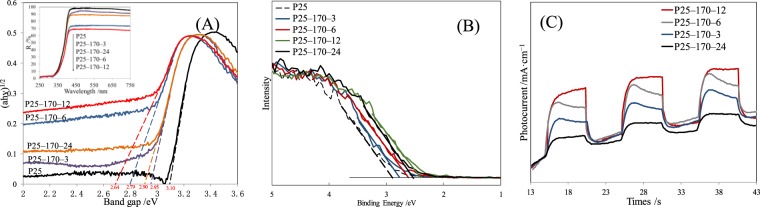
(**A**) The plots of (αhν)^1/2^ versus photon energy calculated from the UV–vis diffuse reflectance spectra (DRS) of different catalysts. (**B**) XPS valence band spectra of different catalysts. (**C**) Transient photocurrent responses of different catalysts under visible light irradiation and dark exposure.

The visible light response of reduced P25 was first characterized by UV–Vis DRS and the results are gathered in Fig. 7(A). The corresponding plots of (*ahν*)^1/2^ versus band gap obtained from DRS are also included in Fig. 7(A). After alcohol solvothermal reduction, *E*_*g*_ of reduced P25 significantly decreased, especially those of P25-6 and P25-12 (2.79 and 2.64 eV). This indicated obvious visible light absorbance. Figure 7(B) depicts the XPS valence band (VB) spectra of pristine P25 and different reduced P25 photocatalysts. Up–shifts in VB energies of reduced P25 photocatalysts were noticed when compared to pristine P25. This up–shift further confirmed the expanding of visible light response of reduced P25, because of the genenation of Ti^3+^ and disorder–crystalline shell-core structure. The two structures generated mid-gap energy levels within the band gap, declining VB energy and *E*_*g*_ values.

Figure 7(C) shows the changes in photocurrent densities of different photocatalysts recorded as a function of time by switching the visible light ON and OFF at intervals of 5 s. Evident photocurrent response might further be observed in reduced P25 under visible light irradiation, reflecting the effective movements of photogenerated electrons in reduced P25^38^. As reduction time increased, the changes in photocurrent response of reduced P25 photocatalysts became consistent with those of *E*_*g*_ values and VB energy of reduced P25 photocatalysts.

### Phenol degradation with reduced P25 under irradiation of visible light

**Figure 8:**
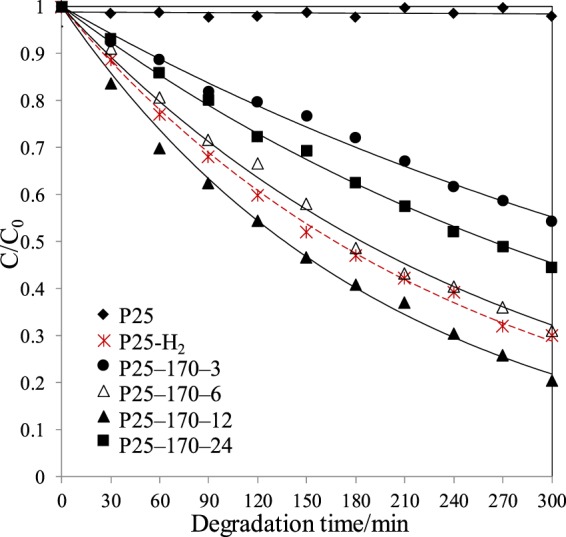
Photodegradation curves of phenol in seawater under visible light irradiation using different photocatalysts.

**Figure 9:**
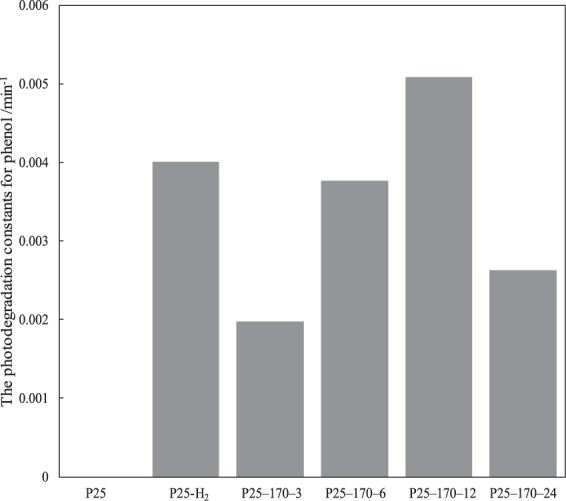
The rate constants of photodegradation by different photocatalysts under visible light irradiation.

The photodegradation curves of phenol in simulated seawater by different catalysts are presented in Fig. 8. Pristine P25 did not show any degradation activity towards phenol under visible light irradiation. By comparison, all reduced P25 photocatalysts depicted photocatalytic activities towards degradation of phenol in seawater irradiated by visible light. The photodegradation process followed first-order reaction kinetics, consistent with the literatures^39,40^. Moreover, the photodegradation rate constants obtained by plotting ln(C_0_/C) as a function of irradiation time *t* are gathered in Fig. 9.

It is also observed from Fig. 9 that among all reduced P25 photocatalysts, P25-170-12 clearly showed the highest photocatalytic activity towards phenol degradation. The photocatalytic activity of P25-170-12 showed a slightly higher performance than P25-H_2_, which caused that many amorphous TiO_2_ in P25-H_2_ acted as deep trapping sites for capturing photogenerated charges. For reduced P25 by alcohol solvothermal process, the conditions were milder than those of hydrogenation reduction. Therefore, only crystalline structure on P25 surface was reduced, leaving internal TiO_2_ crystalline unchanged.

### Optimized reduced P25 catalyst

The reduction conditions during alcohol solvothermal process obviously played key roles in introduction of both Ti^3+^ and disorder–crystalline shell–core structures into reduced P25 photocatalysts, affecting their visible light responses and photocatalytic performances under visible light irradiation. In addition to reduction time, reduction temperature would have an effect. Hence, alcohol solvothermal reduction of P25 was performed under different temperatures for 12 h. As reduction temperature increased to 180 °C, GC chromatography revealed generation of acetic acid in alcohol after solvothermal process, except acetaldehyde (Fig. S15 and Table S6). This was induced by stronger reduction of P25 by alcohol as reduction temperature rose. Therefore, HRTEM showed more evident disorder–crystalline shell–core structures in P25-180-12 catalysts (Fig. S16), with Ti^3+^ contents higher than that in P25-170-12 (Table 2).

**Table 2:** Percentages of Ti valence content in different catalysts.

Catalyst	Reduction Temperature/°C	Reduction Time /h	Content of Ti^4+^/%	Content of Ti^3+^
P25-170-12	170	12	91.2	8.8
P25-180-12	180	12	89.8	10.2
P25-190-12	190	12	90.7	9.3

**Figure 10:**
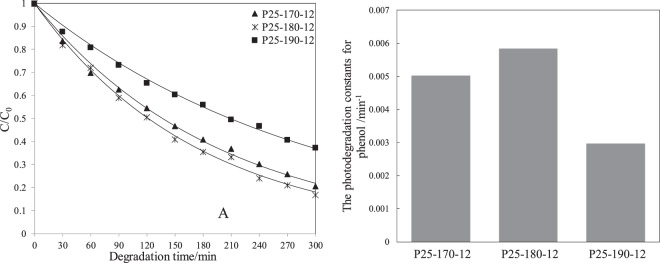
(**A**) Photodegradation curves for phenol under visible light irradiation in seawater by different reduced P25 catalysts; (**B**) The corresponding photodegradation constants.

However, some amorphous TiO_2_ recrystallized into TiO_2_ as reduction temperature increased to 190 °C, declining disorder–crystalline structure and Ti^3+^ content in P25-190-12 (Fig. S16 and Table 2). The recrystallization of amorphous TiO_2_ to crystal TiO_2_ was further confirmed by XRD, in which crystalline diffraction peaks of P25-190-12 became stronger than those of P25-180-12 (Fig. S17). The changes in both disorder–crystalline structure and Ti^3+^ content also affected the visible light responses and photocatalytic performances of the catalysts under visible light irradiation (Figs 10 and S18). With evident disorder–crystalline structure and highest Ti^3+^ content, P25-180-12 showed the best photocatalytic activity among all three reduced P25 photocatalysts under visible light irradiation. Hence, P25-180-12 was selected as candidate for subsequent loading on acrylic hollow spheres.

### Photocatalytic performance of floating hollow spheres irradiated by visible light

P25-180-12 was loaded on inner and outer surfaces of acrylic hollow spheres using silicone coupling agent KH570. Fig. S19 shows the changes in powder containing suspension after centrifugation as a function of reaction time. Only small amounts of powder remained in the suspension after 5 h (0.47 wt% solid content), suggesting that most of powder was loaded on inner and outer surfaces of hollow spheres catalyst (more than 99%). The SEM images of floating spheres further demonstrated the successful loading of P25-180-12 powder catalyst on the spheres (Fig. S20). In SEM images, the large number of small white highlighted morphologies represented the powder catalyst.

**Figure 11:**
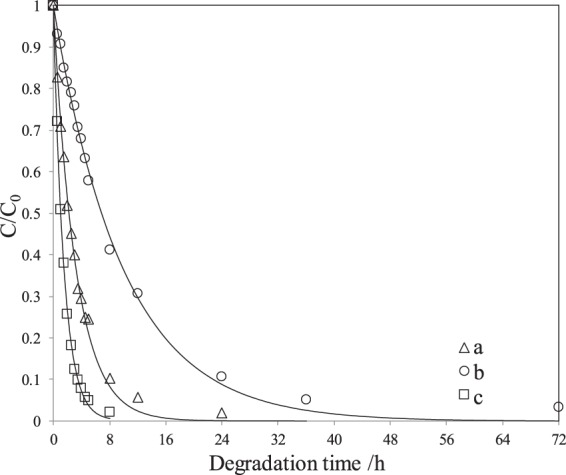
Photodegradation curves of phenol at different concentrations in seawater by floating spheres loaded with P25-180-12 under visible light irradiation. Initial concentration of phenol /mg·L^−1^: (**a**) 5.0, (**b**) 2.0, and (**c**) 1.0.

Compared to Fig. 10, the performances of floating spheres looked weaker than those obtained with powder P25-180-12, as shown in Fig. 11. To confirm this, the photodegradation for phenol in seawater by powder P25-180-12 under the same conditions was also carried out and the corresponding photodegradation curve was also listed in Fig. S21. The reason for this had to do with properties of loaded P25-180-12 on the spheres, which could not form full dispersion in seawater. Hence, surface of the catalyst in contact with phenol obviously reduced, declining the photocatalytic performances of floating spheres. However, the obtained spheres can still stably degrade phenol in simulated seawater with removal rate reaching 95% after 72 h in simulated seawater containing 5.0 mg·L^−1^ phenol. At low phenol concentration (1.0 mg·L^−1^), the performances of floating spheres under visible light irradiation were significantly high and could completely be degraded within 8 h.

To further examine the photocatalysis for phenol by floating spheres under irradiation of different visible light, we also employed a 100 W Xe-lamp as a simulated sunlight resource to irradiate the photodegradation of phenol by floating spheres and the photodegradation curve was listed in Fig. S22. It is found from Fig. S22 that the photodegradation curves irradiated by the fluorescent lamps and Xe-lamp were close, probably because the negligible effect of the change in visible light resources.

### Stability floating hollow spheres loaded by reduced P25 catalyst

**Figure 12:**
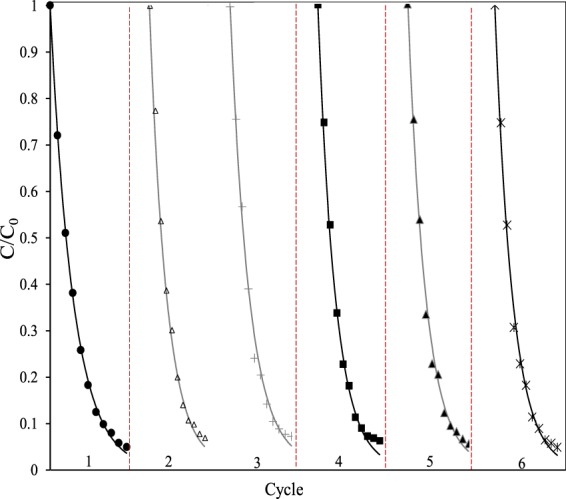
The photodegradation of phenol in seawater performed for six cycles using floating sphered loaded with P25-180-12 under visible light irradiation. The initial concentration of phenol is 1.0 mg·L^−1^.

The stability of floating spheres is vital for practical applications related to removal of organic pollutants from seawater. The reusability of floating spheres was evaluated by six cycle photodegradation experiments for phenol (1.0 mg·L^−1^) in seawater. After each cycle, the floating spheres were recollected and washed with deionized water under ultrasonic treatment followed by drying in vacuum oven at 40 °C before subsequent testing. As shown in Fig. 12, the floating spheres exhibited almost stable activity after six cycles of photodegraded phenol in seawater, highlighting the stability and reusability of the as-prepared floating spheres.

## Conclusions

Commercial P25 photocatalysts were reduced by alcohol solvothermal process to increase their visible light responses. Surface reduction of P25 introduced Ti^3+^ and disorder–crystalline shell–core structures into reduced P25 photocatalysts, enhancing their visible light responses and photocatalytic performances under visible light irradiation. The reduced P25 photocatalysts exhibited high photocatalytic performances towards phenol degradation in seawater under visible light irradiation. As reduction time or temperature of alcohol solvothermal process increased, more Ti^3+^ and shell–core structures were introduced into reduced P25, thereby resulting in higher performances towards phenol degradation in seawater. However, extended time periods and elevated temperatures recrystallized amorphous TiO_2_ in disorder layer, deteriorating the visible light responses and photocatalytic performances of reduced P25 photocatalysts.

The optimized reduced P25 photocatalyst (P25-180-12) was then loaded on inner and outer surfaces of acrylic hollow spheres using silicone coupling agent. The obtained spheres effectively degraded phenol in simulated seawater thanks to fully transparent thin-walled hollow acrylic spheres and high performance reduced P25 photocatalysts. At low phenol concentration (1.0 mg·L^−1^), the performances of floating spheres under visible light irradiation were significantly high with removal rate reaching 95% after 8 h. In addition, the floating spheres highlighted good stability and convenient reusability after six catalytic cycles of phenol photodegradation in seawater, promising for future treatment of organic pollutants in oceans.

## Supplementary information

Supplementary Materials
