# Supplementary material for: Alcohol solvothermal reduction for commercial P25 to harvest weak visible light and fabrication of the resulting floating photocatalytic spheres
Source: Sci Rep. 2019 Sep 25;9:13878. doi: 10.1038/s41598-019-50457-w (PMC6761127; doi:10.1038/s41598-019-50457-w)
Supplement: Supplementary file 1 — Supplementary Materials [file 41598_2019_50457_MOESM1_ESM.docx]

**Alcohol solvothermal reduction for commercial P25 to harvest weak visible light and fabrication of the resulting floating photocatalytic spheres**

Ting Wang, Yao Li, Jia-hao Pan, Yan-ling Zhang, Li–guang Wu*, Chun–ying Dong, Chun-juan Li

School of Environmental Science & Engineering, Zhejiang Gongshang University, Hangzhou, 310012, China

Corresponding author Tel.: +86 571 28008204. Fax.: +86 571 28008215

E–mail address: [wulg64@hotmail.com](mailto:wulg64@hotmail.com) (Li–guang Wu)


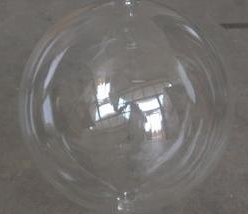


Fig. S1 The digital photo of transparent Acrylic hollow sphere


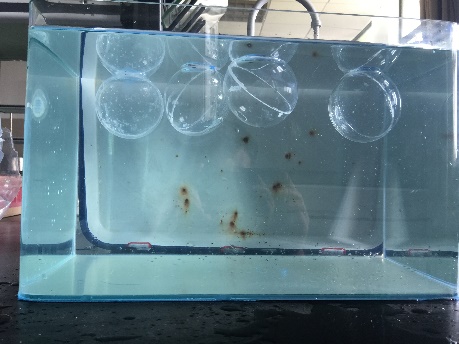

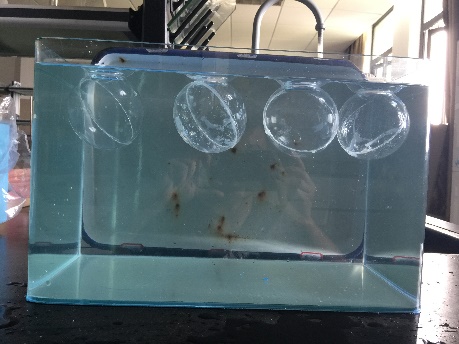

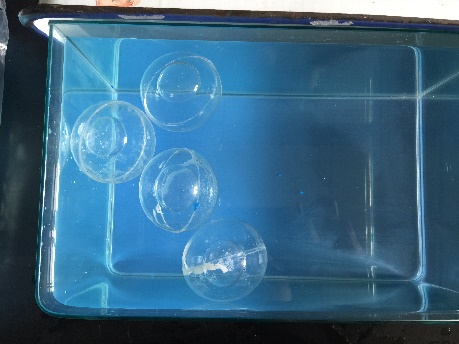


c

b

a

Fig. S2 The digital photos of the photocatalytic spheres floating in the seawater

a. Floating for 24 h; b. Floating for 3 days; c. Floating for 3 days (Top view)

Fig. S3 FTIR spectra of different photocatalysts

a. P25; b. P25-H_2_; c. P25–170–3; d. P25–170–6; e. P25–170–12; f. P25–170–24


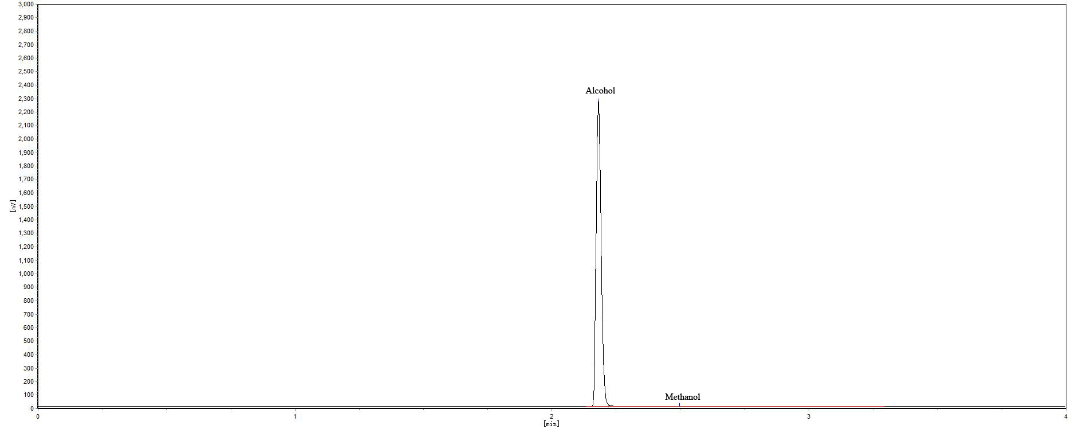


Fig. S4 The chromatogram of solvent before solvothermal process

Table S1 The results from GC for ethanol before solvothermal process

| Number | Component name | Peak height /μV | Peak area /μV·S | Area percentage /% | Mass percentage /% |
| --- | --- | --- | --- | --- | --- |
| 1 | Alcohol | 2259278 | 3088297 | 99.98288 | 99.98288 |
| 2 | Methanol | 408 | 529 | 0.01712 | 0.01712 |


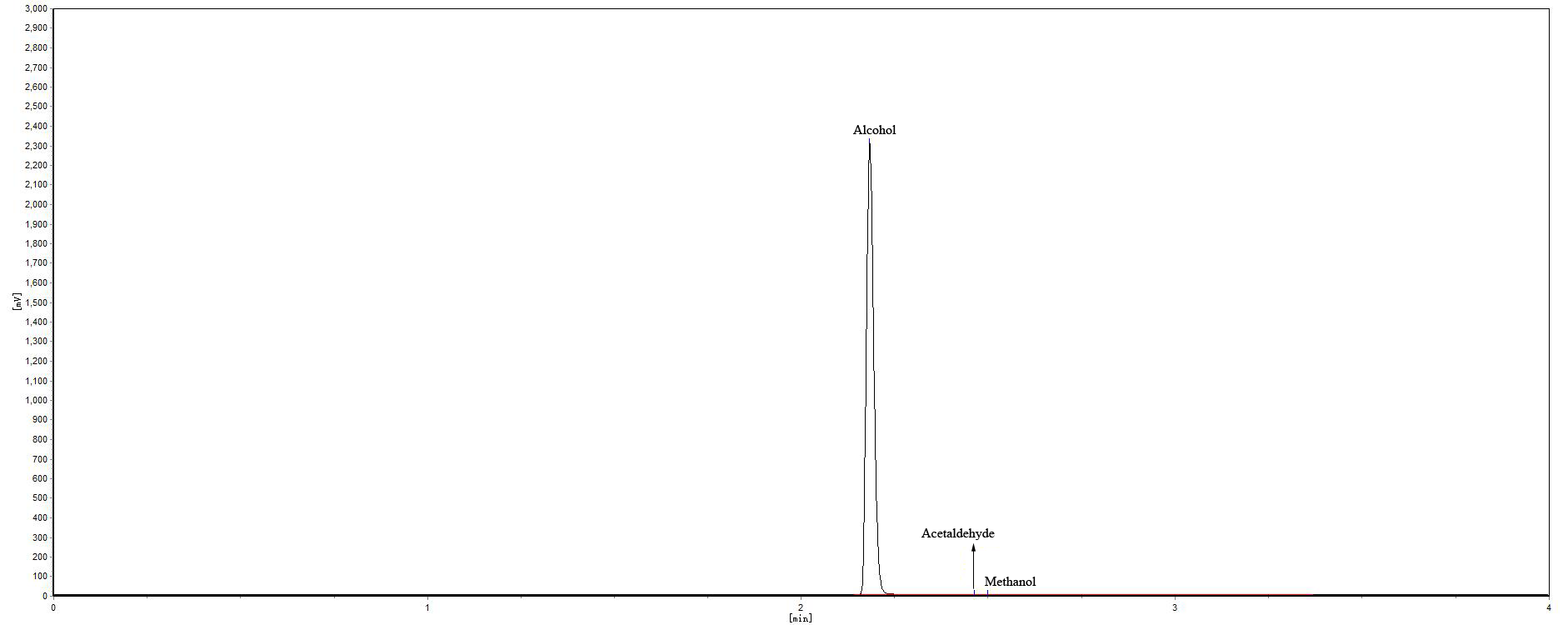


Fig. S5 The chromatogram of solvent after solvothermal process at 170 ºC for 3 h

Table S2 The results from GC for solvent after solvothermal process at 170 ºC for 3 h

| Number | Component name | Peak height /μV | Peak area /μV·S | Area percentage /% | Mass percentage /% |
| --- | --- | --- | --- | --- | --- |
| 1 | Alcohol | 2304444 | 3189011 | 99.983758 | 99.983758 |
| 2 | Acetaldehyde | 194 | 251 | 0.00788 | 0.00788 |
| 3 | Methanol | 284 | 377 | 0.01183 | 0.01183 |


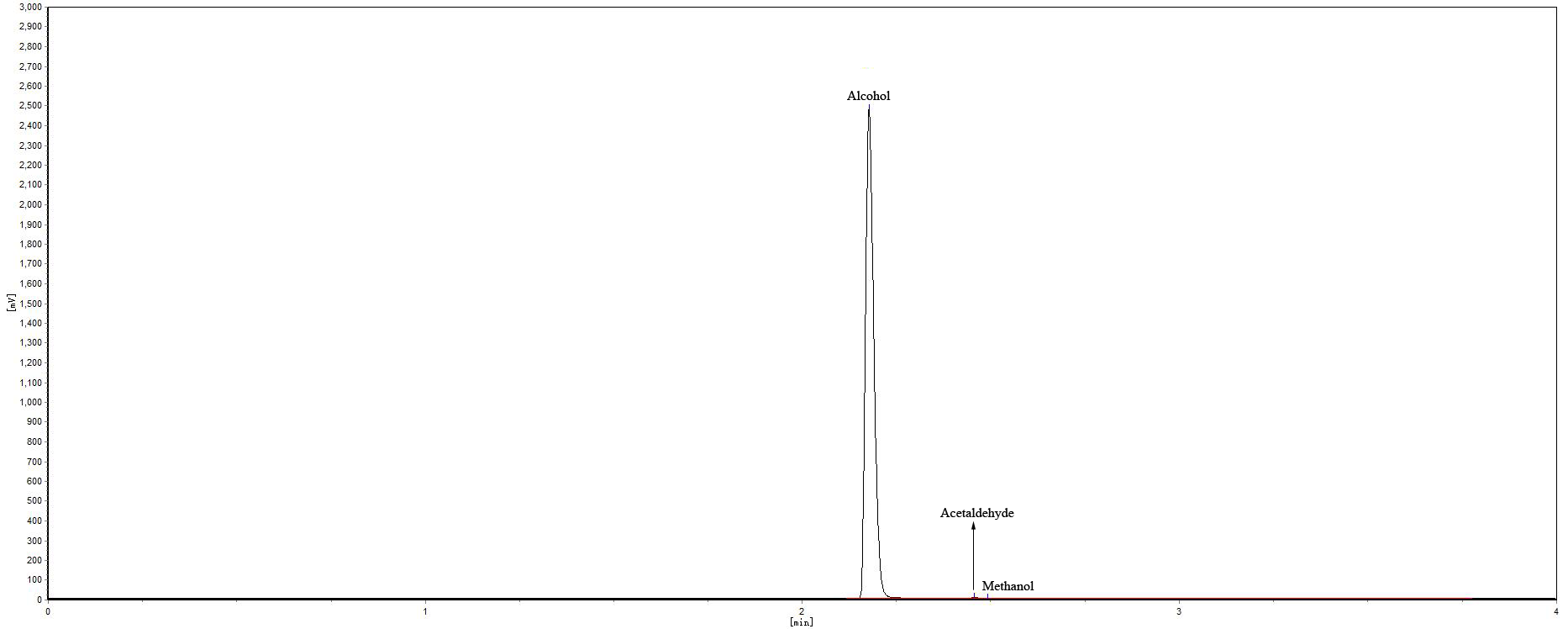


Fig. S6 The chromatogram of solvent after solvothermal process at 170 ºC for 6 h

Table S3 The results from GC for solvent after solvothermal process at 170 ºC for 6h

| Number | Component name | Peak height /μV | Peak area /μV·S | Area percentage /% | Mass percentage /% |
| --- | --- | --- | --- | --- | --- |
| 1 | Alcohol | 2476950 | 3824683 | 99.83758 | 99.83758 |
| 2 | Acetaldehyde | 3960 | 5497 | 0.14348 | 0.14348 |
| 3 | Methanol | 598 | 726 | 0.01894 | 0.01894 |


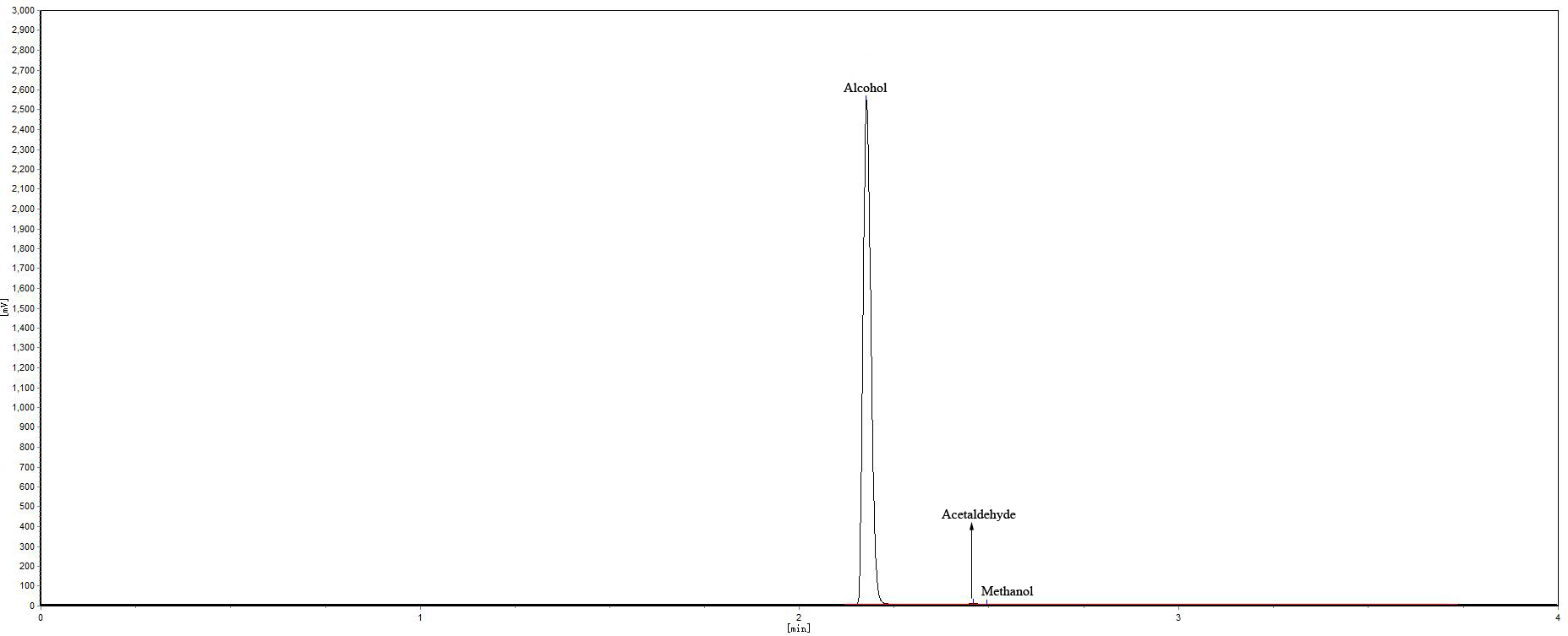


Fig. S7 The chromatogram of solvent after solvothermal process at 170 ºC for 12 h

Table S4 The results from GC for solvent after solvothermal process at 170 ºC for 12 h

| Number | Component name | Peak height /μV | Peak area /μV·S | Area percentage /% | Mass percentage /% |
| --- | --- | --- | --- | --- | --- |
| 1 | Alcohol | 2542588 | 3685688 | 99.74833 | 99.74833 |
| 2 | Acetaldehyde | 6360 | 8413 | 0.22768 | 0.22768 |
| 3 | Methanol | 714 | 886 | 0.02399 | 0.02399 |


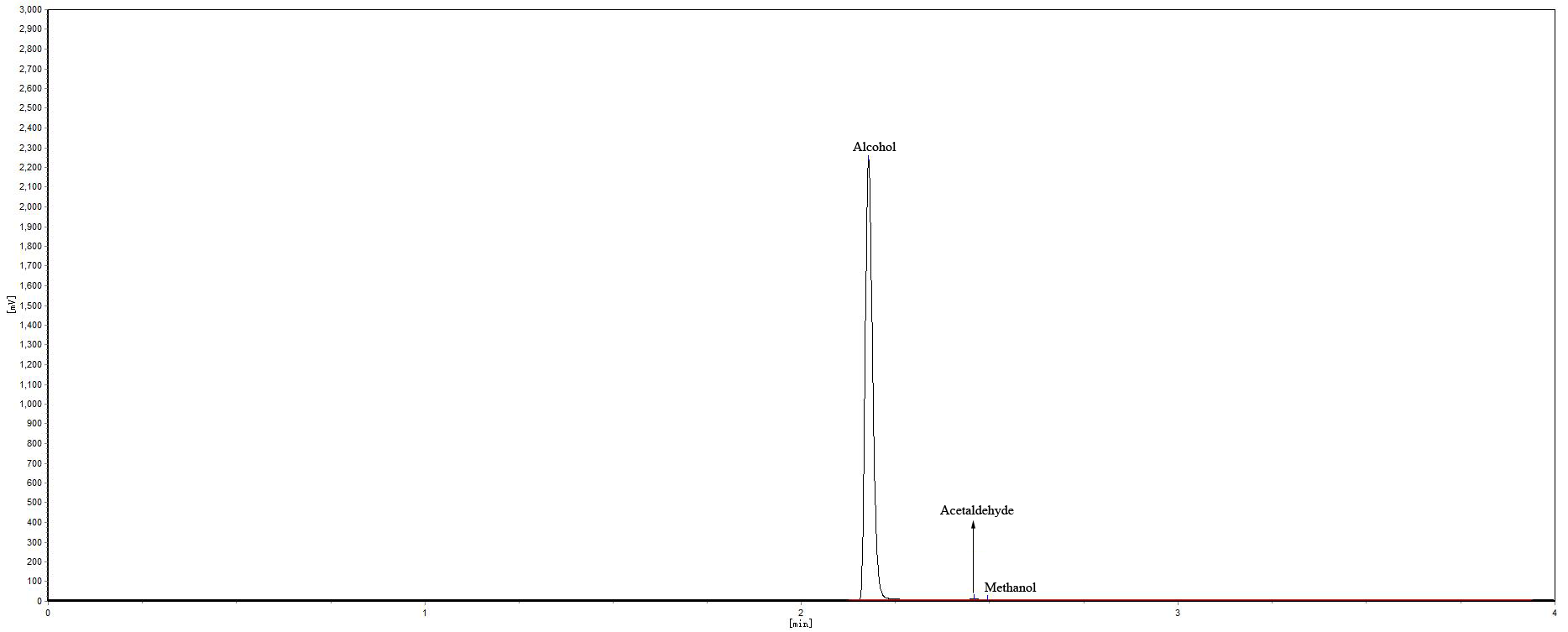


Fig. S8 The chromatogram of solvent after solvothermal process at 170 ºC for 24 h

Table S5 The results from GC for solvent after solvothermal process at 170 ºC for 24 h

| Number | Component name | Peak height /μV | Peak area /μV·S | Area percentage /% | Mass percentage /% |
| --- | --- | --- | --- | --- | --- |
| 1 | Alcohol | 2230068 | 3115296 | 99.77217 | 99.77217 |
| 2 | Acetaldehyde | 4844 | 6375 | 0.20417 | 0.20417 |
| 3 | Methanol | 610 | 739 | 0.02366 | 0.02366 |















d

c

b

a

f

e

Fig. S9 TEM images of different photocatalysts

a. P25; b. P25-H_2_; c. P25–170–3; d. P25–170–6; e. P25–170–12; f. P25–170–24


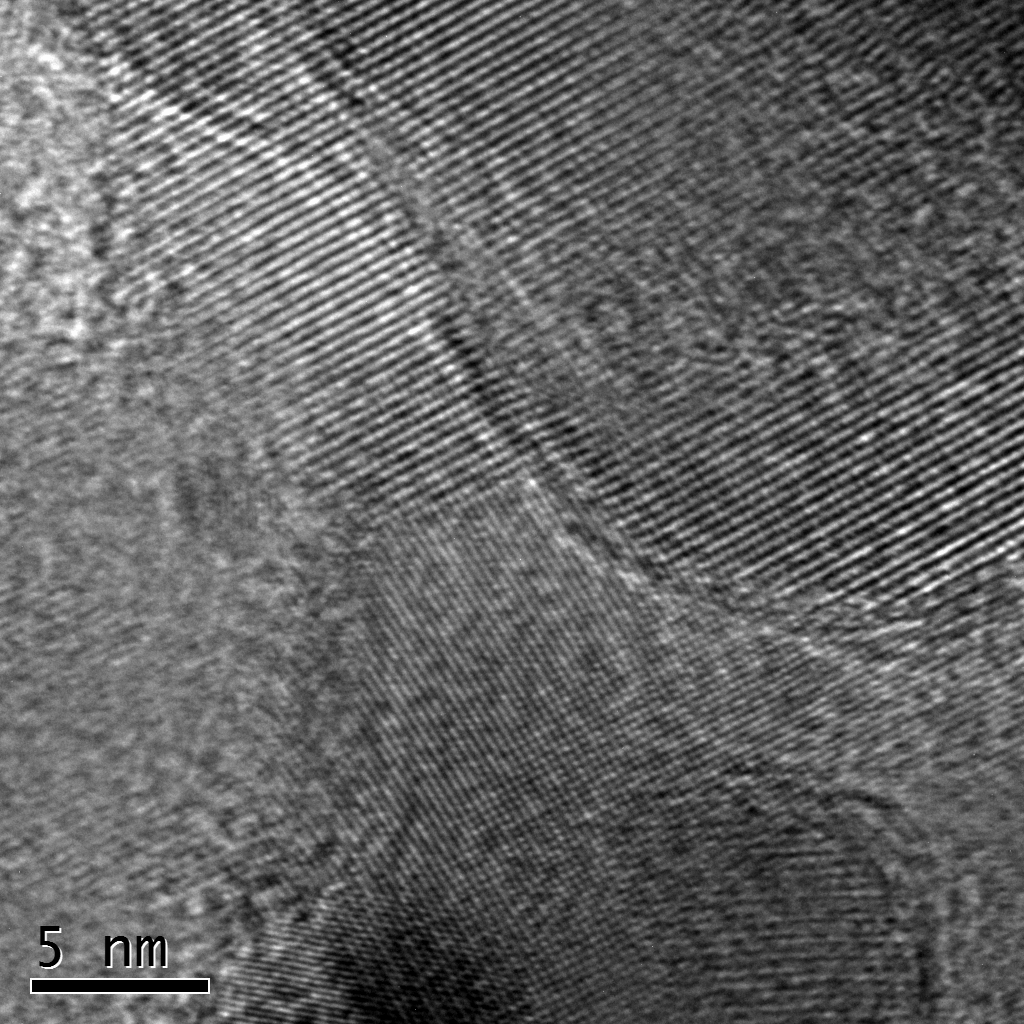


Fig. S10 HRTEM image of pristine P25 photocatalyst




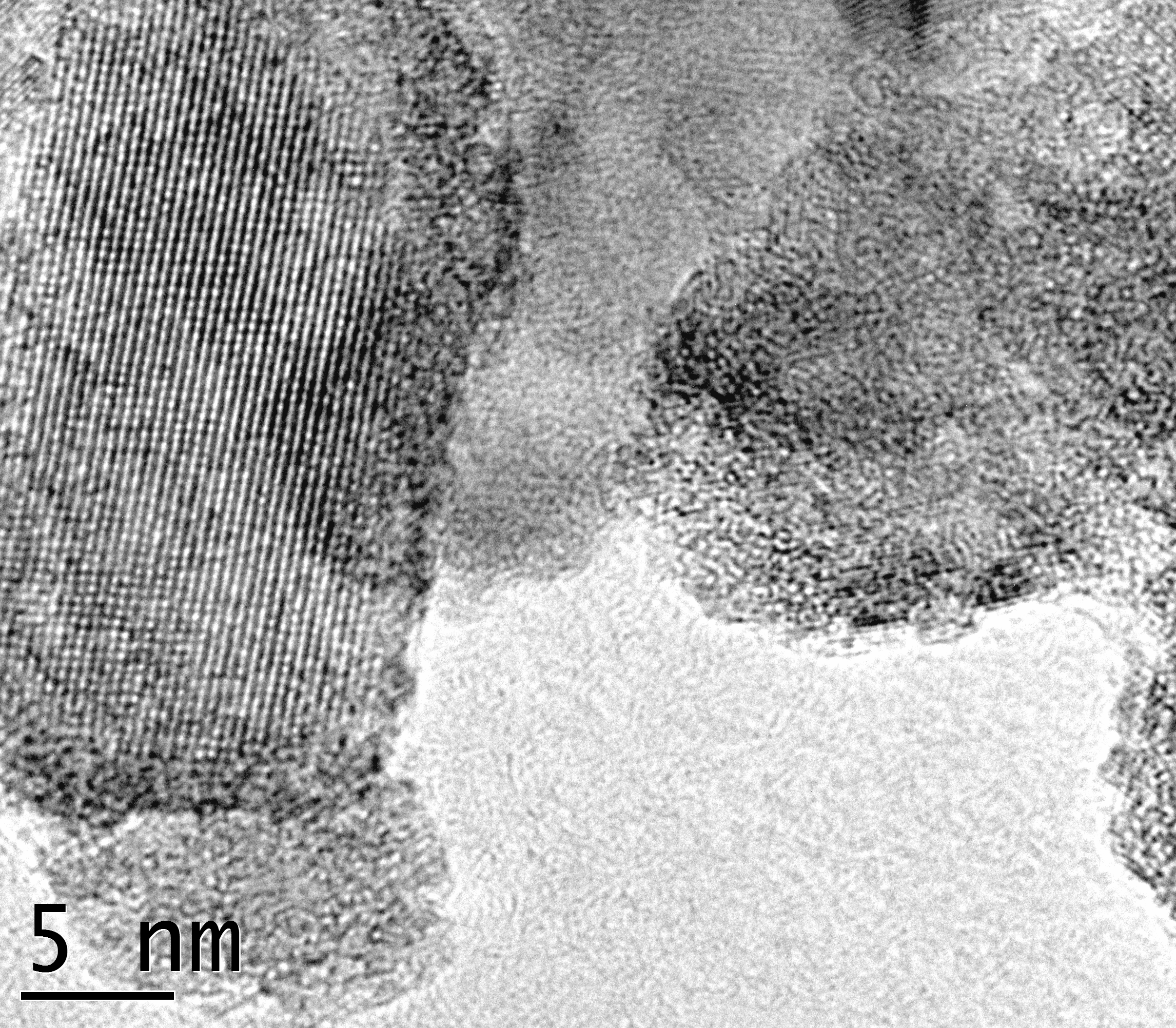


Fig. S11 HRTEM image of reduced P25 photocatalyst by hydrogenation reduction for 20 days

Fig. S12 XRD patterns of different photocatalysts

a. P25; b. P25-H_2_; c. P25–170–3; d. P25–170–6; e. P25–170–12; f. P25–170–24

Fig. S13 XPS profiles of different photocatalysts

a. P25; b. P25-H_2_; c. P25–170–3; d. P25–170–6; e. P25–170–12; f. P25–170–24

c

b

a

Fig. S14 XPS profiles for Ti 2p in different reduced photocatalysts

a. P25–170–3; b. P25–170–6; c. P25–170–24


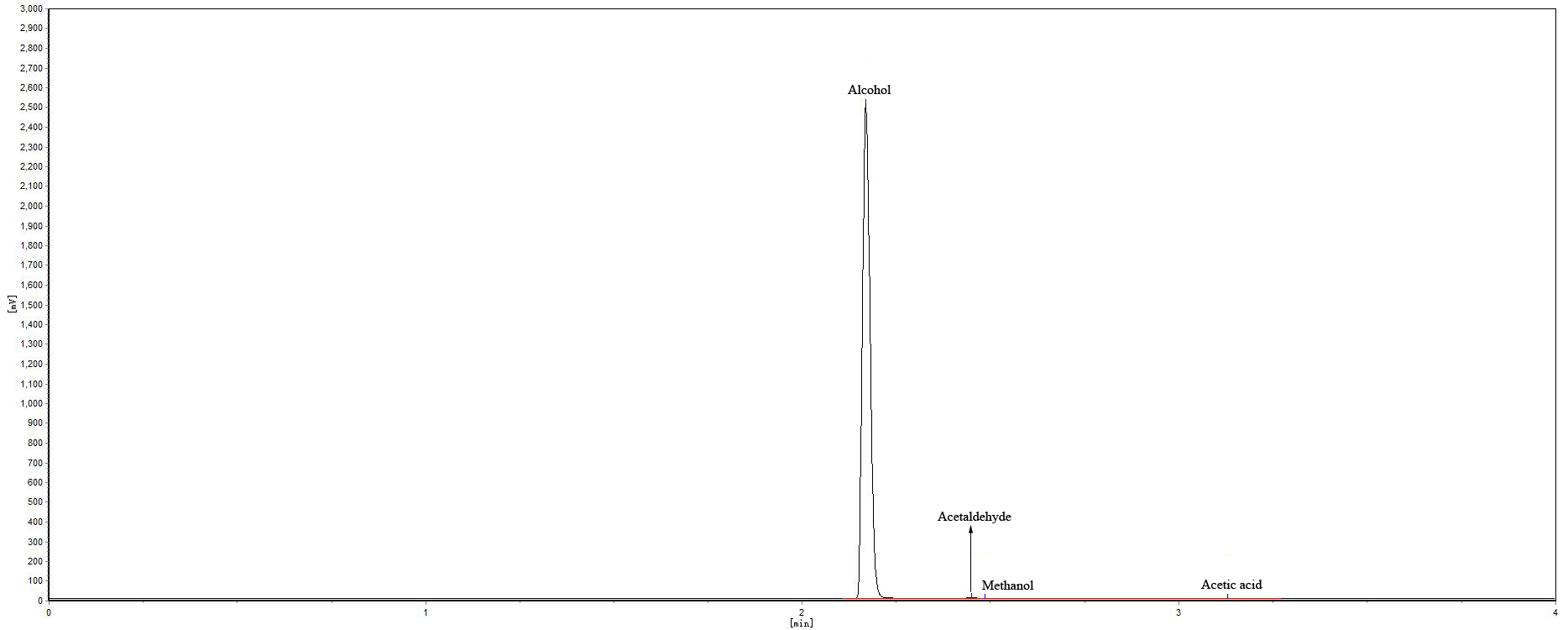


Fig. S15 The chromatogram of solvent after solvothermal process at 190 ºC for 6 h

Table S6 The results from GC for solvent after solvothermal process at 190 ºC for 6 h

| Number | Component name | Peak height /μV | Peak area /μV·S | Area percentage /% | Mass percentage /% |
| --- | --- | --- | --- | --- | --- |
| 1 | Alcohol | 2505272 | 3538583 | 99.81792 | 99.81792 |
| 2 | Acetaldehyde | 4112 | 5431 | 0.15319 | 0.15319 |
| 3 | Methanol | 636 | 807 | 0.02278 | 0.02278 |
| 4 | Acetic acid | 120 | 217 | 0.00611 | 0.00611 |


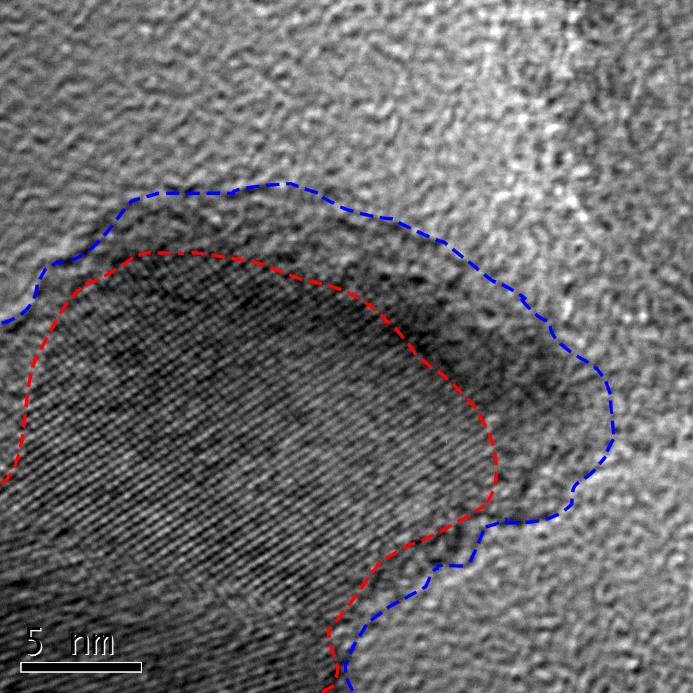

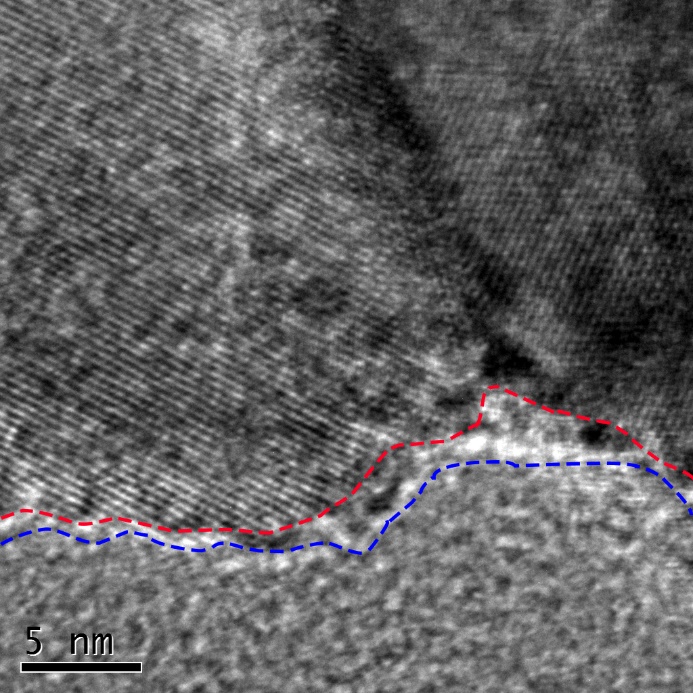


a

b

Fig. S16 HRTEM images of P25–180–12 and P25–190–12

a. P25–180–12; b. P25–190–12

Fig. S17 XRD patterns of pristine P25 and reduced P25 by hydrothermal process under different temperatures

a. pristine P25; b. P25–170–12; c. P25–180–12; d. P25–190–12

Fig. S18 The plots of (αhν)^1/2^ vs. photon energy calculated from UV–vis diffuse reflectance spectra of different catalysts

(The inset picture is the corresponding UV–vis diffuse reflectance spectra)

a. pristine P25; b. P25–170–12; c. P25–180–12; d. P25–190–12


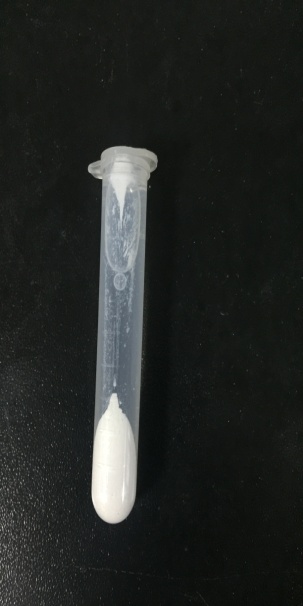

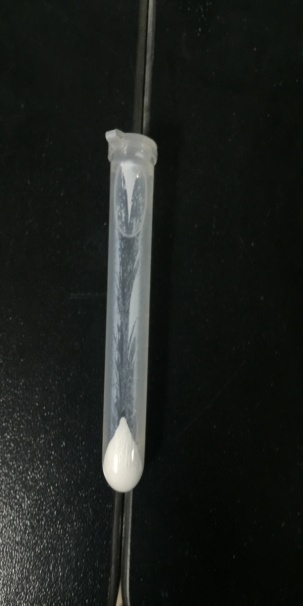

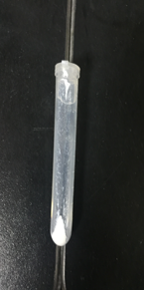


C

B

A

Fig. S19 The reaction solution after centrifugation during preparation of floating photocatalytic spheres

Reaction time /h: A. 0; B. 3; C. 5


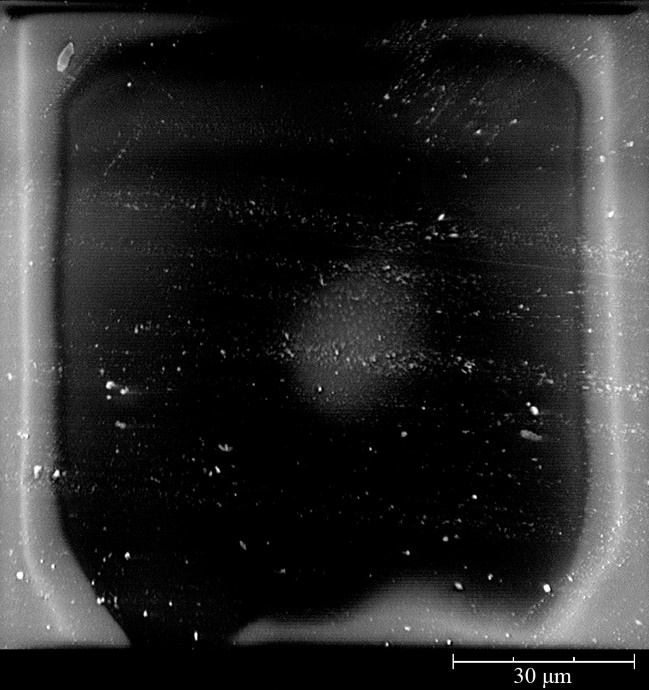

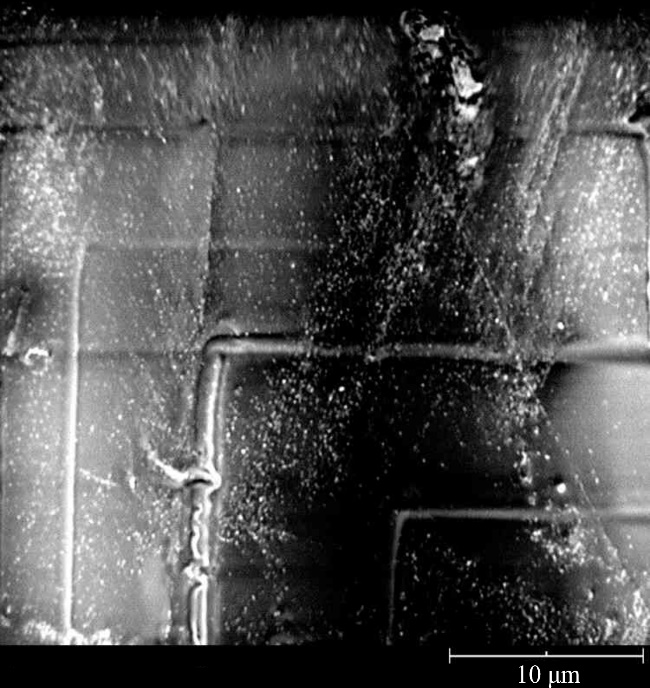


Fig. S20 SEM images of the floating photocatalytic sphere loading with P25–180–12

Fig. S21 Photodegradation curves of phenol in seawater by floating spheres and powder P25-180-12 under visible light irradiation. Initial concentration of phenol: 5.0 mg·L^−1^

Fig. S22 Photodegradation curves of phenol in seawater by floating spheres under visible light irradiation and simulated sunlight. Initial concentration of phenol: 5.0 mg·L^−1^
